# Preservation-to-Precision in Severe Early Childhood Caries: A Narrative Review of Silver Diamine Fluoride—When “Buying Time” Must Not Become “Selling Time”

**DOI:** 10.3390/ijerph23050656

**Published:** 2026-05-14

**Authors:** Ziad D. Baghdadi

**Affiliations:** 1 Centre for Community Oral Health, Dr. Gerald Niznick College of Dentistry, University of Manitoba, Winnipeg, MB R3B 0L8, Canada; drziadeddin.albaghdadi@gmail.com or zalbaghdadi@atsu.edu; 2 Division of Pediatric Dentistry, Department of Preventive Dental Sciences, Dr. Gerald Niznick College of Dentistry, University of Manitoba, Winnipeg, MB R3E 0W2, Canada; 3 TopSmiles Pediatric Dentistry and Orthodontics, Winnipeg, MB R2M 3A4, Canada; 4 Butterfly Dental Group, Winnipeg, MB R3Y 1P5, Canada

**Keywords:** severe early childhood caries, silver diamine fluoride, minimally invasive dentistry, interim stabilization, definitive restorative care, pediatric dental access, health equity, children’s rights, preservation-to-precision

## Abstract

**Highlights:**

**Public health relevance—How does this work relate to a public health issue?**

- Severe early childhood caries (SECC) affects preschool children worldwide, with consequences for child health, family functioning, and health systems. The use of silver diamine fluoride (SDF) as an interim measure has expanded due to persistent barriers to restorative care.
- This review examines the public health tension between using SDF to arrest lesions and the risk of normalizing temporary treatment as a substitute for timely definitive care in high-income countries.

**Public health significance—Why is this work of significance to public health?**

- Many SDF-treated primary teeth need further care within approximately 2 years. Delays in sedation or anesthesia usually last weeks to months, not years. This challenges the idea that SDF alone maintains teeth until they fall out naturally.
- The proposed “preservation-to-precision” framework is ethically grounded and child-centered, separating interim stabilization from long-term care, setting clear exit criteria, and considering health equity.

**Public health implications—What are the key implications or messages for practitioners, policy makers and/or researchers in public health?**

- Practitioners should document whether SDF is used based on disease stage or due to a delay in definitive care, and set monitoring intervals and exit criteria rather than open-ended temporization.
- Policy makers must address barriers to operating room access and other system issues that can shift interim care from a deliberate bridge to an unplanned default. “Buying time” should help children access timely, definitive care, not prolong exposure to disease.

**Abstract:**

Severe early childhood caries (SECC) in preschool children is a progressive, multifactorial disease with far-reaching consequences for child health, family functioning, and health systems. Minimally invasive dentistry (MID), particularly 38% silver diamine fluoride (SDF), is increasingly used to arrest lesions and “buy time” when definitive restorative care is delayed. This narrative review synthesizes current evidence-based guidelines and real-world utilization data to clarify the appropriate role and limits of SDF in SECC management. Professional guidance supports SDF for lesion arrest within an ongoing caries management plan, but does not endorse it as a universal long-term substitute for durable restorative care. Observational studies show that many SDF-treated primary teeth receive additional intervention within approximately 2 years, and any delay in sedation/general anesthesia is typically measured in weeks to months. A large recent private practice study found that 35% of children with caries progressed to higher-intensity treatment (restoration or extraction) over a median of 547 days, reinforcing the time-limited nature of interim stabilization. We propose a “preservation-to-precision” framework that prioritizes child-centered outcomes—freedom from pain and infection, durable function, and acceptable psychosocial impact—through risk-based, tooth- and child-specific planning, realistic follow-up assessment, and clear exit criteria for transition to definitive care. In high-income settings, the ethical value of “buying time” depends on whether systems use that time to advance children toward timely, definitive care rather than normalizing prolonged temporization as routine practice.

## 1. Introduction

Severe early childhood caries (SECC) in preschool children is not merely a localized dental condition. It is a preventable, progressive disease that can impair sleep, nutrition, growth, development, family functioning, and oral health-related quality of life [1]. The American Academy of Pediatric Dentistry (AAPD) defines SECC with clear clinical thresholds and links early caries burden to downstream harms, including emergency department visits, hospitalizations, high treatment costs, lost school days, and diminished quality of life [1].

In recent years, minimally invasive dentistry (MID), particularly 38% silver diamine fluoride (SDF), has gained prominence as a strategy to arrest lesions and “buy time” when definitive restorative care is not immediately feasible. Professional guidelines support SDF for arresting cavitated lesions in primary teeth when used as part of an ongoing caries management plan within a dental home [2,3,4,5,6]. However, real-world use has sometimes extended beyond these evidence-supported boundaries, raising concerns that interim stabilization may inadvertently become a long-term substitute for definitive restorative care in children whose teeth must remain functional for several years.

### 1.1. Epidemiology and Burden of SECC

Early childhood caries affects an estimated 530 million children worldwide, with SECC representing the most severe end of the spectrum. In high-income countries, the disease remains prevalent among disadvantaged populations, and hospital-based treatment under general anesthesia (GA) constitutes a major health system cost. Access to timely GA is increasingly constrained, leading to growing reliance on interim strategies such as SDF [7,8,9,10,11,12].

### 1.2. Role of SDF in Current Practice

SDF is effective at arresting dentin caries, with systematic reviews reporting arrest rates of 60–80% over 12–24 months [8,9,10,11]. Its ease of application and the absence of local anesthesia or drilling have made it attractive in settings where behavioral or systemic barriers limit restorative care. However, lesion arrest is not equivalent to restoring form, function, or aesthetics, and many treated teeth eventually require additional intervention.

### 1.3. The Tension Between Interim and Definitive Care

A critical but underexamined question is whether SDF is used as a deliberate, time-limited bridge to definitive care or as an unplanned default due to persistent access barriers. In high-income countries, operating room (OR) denials and long waiting lists have been documented, raising the risk that “buying time” becomes “selling time”—i.e., prolonged temporization without a pathway to durable health outcomes.

### 1.4. Rationale and Novelty of This Review

This review builds on our prior clinical framework for SDF exit criteria, but shifts focus to public health ethics and systems-level analysis. Specifically, we aim to (i) summarize what evidence-based guidelines support regarding SDF in SECC; (ii) critically appraise real-world data on SDF outcomes and utilization patterns, including a newly published large private-practice study; (iii) present the “preservation-to-precision” framework as an ethically grounded, child-centered approach; and (iv) discuss how system constraints in high-income settings can transform interim care into de facto long-term management. No prior review has systematically addressed this ethical and systems risk.

**Why this review is needed now:** Despite evidence-based guidelines supporting SDF for lesion arrest, real-world practice has extended its use beyond these boundaries, often without explicit exit criteria or a clear pathway to definitive care. This gap between evidence and practice has grown as access barriers to operating room and sedation services have worsened in many high-income countries. Recent large-scale private practice data [13] now provide, for the first time, real-world escalation rates and time-to-event outcomes that can inform ethical and systems-level guidance. No prior review has systematically examined the risk that “interim stabilization” becomes a default long-term substitute for restorative care, nor has any proposed a child-centered, equity-focused framework to separate bridging from prolonged temporization. The present review addresses this gap by synthesizing guideline recommendations, real-world utilization patterns, and ethical principles into a practical preservation-to-precision pathway.

## 2. Methods

This narrative review is based on a systematic search of PubMed and the Cochrane Library for English-language publications from 2015 to 2026, using the terms “severe early childhood caries,” “silver diamine fluoride,” “minimally invasive dentistry,” “interim stabilization,” and “pediatric dental access.” Additional sources were identified from the reference lists of included articles and from policy documents of the American Dental Association (ADA) and the AAPD. The authors selected prioritized evidence-based guidelines, systematic reviews, and large observational studies reporting clinically meaningful outcomes (lesion arrest, subsequent treatment, time to sedation/GA, tooth survival) in children with SECC.

**Selection rationale:** Among available real-world studies, we prioritized those with (i) sample sizes > 100 children, (ii) follow-up duration ≥ 12 months, (iii) reporting of clinically meaningful endpoints (subsequent treatment, time to sedation/GA, tooth survival), and (iv) explicit adjustment for case mix or propensity matching when available. Studies limited to satisfaction surveys or short-term (<6 months) arrest rates were excluded to focus on outcomes relevant to the “bridge vs. default” question.

After applying the inclusion criteria (sample size > 100 children, follow-up duration ≥ 12 months, reporting of clinically meaningful endpoints such as subsequent treatment, time to sedation/GA, or tooth survival, and exclusion of studies limited to satisfaction surveys or <6-month arrest rates), six primary studies were included for detailed analysis: Davis et al. (2020) [14], Schlotz et al. (2024) [15], Meyer et al. (2023) [16], Raskin et al. (2021) [17], and Whitlatch et al. (2026) [13], plus the guideline documents from ADA and AAPD [2,3,4,5,6]. The follow-up durations across these studies ranged from 1 year (Davis) to approximately 7 years (Whitlatch et al., 2026) [13], with a median follow-up of 1.75 years in the largest cohort (*n* = 8857). The search time frame covered publications from January 2015 through April 2026. Additional inclusion criteria required that studies provide separate data for SDF-treated primary teeth (not mixed permanent dentition) and that they report either event-time outcomes (e.g., median days to sedation/GA) or proportion of teeth/children requiring additional intervention within a defined period. Studies that reported only arrest rates without longitudinal follow-up for subsequent treatment were excluded, as were case series with <100 participants.

### Characteristics of Included Study Populations

The primary studies selected for this review *(n* = 6) encompassed the following population groups:

- **Age range:** All studies included children with primary dentition, with a specific focus on ages 1–6 years. The Meyer et al. (2023) [16] and Whitlatch et al. (2026) [13] studies reported mean ages of 3.8–4.2 years. Schlotz et al. (2024) [15] analyzed claims for children aged 1–5 years at the time of SDF application.
- **Clinical settings:** Davis et al. (2020) [14] and Whitlatch et al. (2026) [13] were conducted in large, multi-site private pediatric dental practices in the United States. Raskin et al. (2021) [17] used data from community-based safety-net dental clinics. Schlotz et al. (2024) [15] analyzed private dental insurance claims from a mixed payer population (commercial insurance and some Medicaid).
- **Geographic distribution:** All studies originated in the United States, limiting generalizability to other high-income countries with different healthcare systems (e.g., England and Australia). International comparisons are drawn from separate health system reports (referenced in Section 6.2).
- **Socioeconomic proxies:** In claims-based studies [15], private insurance was the main payer, indicating a predominantly middle-income population. In safety-net clinic studies [17], the majority of children were from low-income families eligible for public assistance. Whitlatch et al. [13] did not report payer status but described a private practice setting with a mix of insurance types.
- **Clinical severity:** All studies required cavitated dentin lesions consistent with SECC. Whitlatch et al. [13] further stratified children by caries severity class (I–V), with detailed escalation rates for each subtype (e.g., Class III and V, 42–50% escalation).
- **Limitations:** No included study provided stratified data by race/ethnicity, rural vs. urban residence, or Indigenous populations. This represents an important research gap for health equity analyses.

## 3. The Preservation-to-Precision Framework

Preservation-to-precision is a clinical and ethical framework for managing 3- to 4-year-olds with SECC. It is not proposed as a new guideline but as a lens to help clinicians and systems align decisions about SDF and MID with child-centered endpoints: freedom from pain and infection, durable function, and acceptable psychosocial impact.

**Definition of definitive restorative care in this Review:** Restoration of form, function, and aesthetics to a level expected to remain durable for the remaining lifespan of the primary tooth (typically > 2 years for a 3- to 4-year-old) without the need for re-intervention due to material failure, recurrent caries, or loss of marginal integrity. Examples include stainless steel crowns, multi-surface composites with appropriate isolation, and preformed crowns. Interim stabilization (including SDF alone or temporary restorations) does not meet this definition.

**Preservation** is broader than “avoiding the drill.” It includes conserving tooth structure when appropriate, as well as preserving the child’s comfort and emotional safety, the family’s capacity to follow through with care, and the child’s opportunity for a stable health trajectory rather than recurrent crisis [1]. Preservation does not mean doing less dentistry; it means minimizing iatrogenic harm without minimizing the child’s lived burden of disease.

**Precision** means tailoring the intervention to the tooth-level condition, the child’s overall context, and the system’s capacity to deliver timely care. Caries management frameworks, such as the International Caries Classification and Management System (ICCMS), emphasize staging disease, assessing risk, and selecting nonoperative care for early lesions and conservative operative care for more extensive lesions, with the goal of achieving health outcomes rather than simply performing procedures [7].

In practical terms, precision requires explicit decisions on the following:

- Tooth-level prognosis and expected service time;
- Disease severity and symptom status;
- Follow-up feasibility and caregiver capacity;
- Behavioral and care-setting needs;
- Clear “exit criteria” for interim stabilization (e.g., symptom triggers, progression indicators, or timeline thresholds).

This framework distinguishes two questions often conflated in SECC discourse: (1) can SDF arrest lesions (supported by evidence), and (2) should interim stabilization be treated as a universal long-term pathway for severe disease (a stronger claim that requires more careful framing)?

## 4. What Evidence-Based Guidelines Support—And Do Not Establish

### 4.1. Nonrestorative Therapies, Including SDF

The ADA evidence-based clinical practice guidelines on nonrestorative treatments support 38% SDF as an effective option for arresting cavitated caries lesions in primary teeth, along with sealants and other fluoride-based therapies, when used within an ongoing caries management plan [2]. Similarly, the AAPD states that 38% SDF is effective for arresting cavitated lesions and emphasizes its use within a dental home [3,4,5,6].

Systematic reviews and meta-analyses have consistently reported that SDF arrests dentin caries in primary teeth, with higher arrest rates observed with repeated applications; however, they also highlight heterogeneity in protocols, outcome definitions, and follow-up periods, and underscore that lesion arrest is not equivalent to long-term restoration of form and function in high-burden SECC [8,9,10,11].

### 4.2. Restorative Care

The ADA evidence-based guidelines for restorative treatment support conservative operative strategies for moderate and advanced cavitated lesions, including approaches that limit unnecessary removal of tooth structure while aiming for durable disease control and restoration of function [12]. According to the preservation-to-precision model, “minimal invasiveness” is best understood as an outcome-oriented commitment—minimizing harm while achieving health endpoints—rather than a fixed ceiling on intervention intensity.

### 4.3. What Guidelines Do Not Establish?

Neither the ADA nor the AAPD guidelines endorse SDF as a universal, long-term substitute for durable restorative care for all children with SECC, irrespective of disease severity, tooth prognosis, aesthetic and functional needs, follow-up feasibility, or symptom status [2,3,4,5,6]. The strongest guideline-supported claim is that arresting lesions, rather than guaranteeing multi-year outcomes such as durable function, form, aesthetics, or tooth-level survival to exfoliation, is the best approach in high-burden SECC without subsequent definitive care when indicated.

### 4.4. Consensus on SDF Application Frequency

Neither the ADA nonrestorative guideline [2] nor the AAPD policies [3,4,5,6] specify a standardized frequency for SDF application. Guidelines recommend SDF “as part of an ongoing caries management plan within a dental home” but do not mandate a universal interval (e.g., every 6 months vs. annually).

Systematic reviews provide some empirical guidance but no consensus:

- Worthington et al. (Cochrane 2024) [8] concluded that more frequent applications (e.g., every 6 months) tend to yield higher lesion arrest rates than single applications, but the optimal frequency remains unclear because of heterogeneity in study protocols.
- Chibinski et al. (2017) [10] reported that two or more applications over 12–18 months increased arrest rates by approximately 15–20% compared to a single application.
- Seifo et al. (2019) [9] noted that most included studies used application intervals of 6 to 12 months, with no significant difference in arrest rates between 6-month and 12-month intervals in head-to-head trials (limited evidence).

**Expert opinion and pragmatic recommendations:** In the absence of high-level evidence for a specific interval, many clinical experts recommend reapplying SDF every 6–12 months until either (a) lesion arrest is confirmed and the tooth is close to exfoliation, or (b) definitive restorative care is provided. The preservation-to-precision pathway (Section 7) adopts a pragmatic reassessment interval of 3–6 months for active monitoring, not as a therapeutic frequency recommendation per se. This interval aligns with real-world data showing that most failures or escalations occur within 18 months, making a 6-month recall window reasonable for detecting progression.

**Research gap:** Prospective dose-finding studies comparing SDF application frequencies (e.g., single, annual, or biannual) with long-term outcomes (tooth survival, quality of life, cost-effectiveness) are needed. Until then, clinicians should document and justify their chosen interval based on lesion severity, the child’s risk level, and access to definitive care.

## 5. Real-World Evidence on SDF Utilization and Outcomes

Real-world studies offer valuable insights into how SDF functions in practice, but their observational nature requires careful interpretation. The key findings are summarized in Table 1, which includes a recent large-scale study of a private practice implementation [13].

**Additional findings from Whitlatch et al. [13]:** In a large private pediatric practice (9 locations, *n* = 8857 children with ≥2 visits), disease management procedures (SDF, sealants) were the dominant treatment. Among children with caries, 46% received treatment at their initial visit. The median duration of care from first to final treatment was 637 days, and the median expenditure was $1546 USD. Importantly, children with severe subtypes (Class III and V) had the highest escalation rates (42% and 50%, respectively), underscoring that tooth-level precision triage is essential.

These data support SDF as a meaningful bridge in selected circumstances, but they also indicate that “buying time” should not be equated with achieving long-term exfoliation outcomes, particularly for 3- to 4-year-olds whose primary teeth must function for several years [15]. When SDF delays sedation/GA, the interval is typically weeks to months, underscoring that the value of that time depends on whether it is used to progress toward definitive care [16]. The Whitlatch et al. study [13] added that even in a well-resourced private practice with a standardized protocol, more than one-third of children eventually require escalation, typically about 18 months after initial treatment.

### Aesthetic Considerations and Their Clinical Impact

A well-documented drawback of silver diamine fluoride is the irreversible black staining of arrested carious lesions, caused by the formation of silver oxide. This cosmetic change, while clinically harmless, can be a source of concern for children and caregivers, particularly when anterior teeth are affected.

Systematic reviews and meta-analyses of parental acceptance [9] report that acceptance rates vary widely (36–91%) depending on tooth location, pretreatment counseling, and cultural attitudes toward visible staining. Anterior teeth are significantly less accepted than posterior teeth. In a qualitative study of dental professionals [9], clinicians noted that upfront discussion of staining is essential for informed consent, and that some parents decline SDF entirely for aesthetic reasons.

From an ethical and practical standpoint, the following considerations should guide clinical use:

- Informed consent must explicitly include discussion of black staining, with photographs or examples when possible.
- Tooth location should influence decision-making: for anterior teeth with high aesthetic demands, alternative interim strategies (e.g., SMART, temporary restorations) may be preferred if durable restorative care is delayed.
- Mitigation strategies include applying SDF only to posterior lesions, using SDF under a restoration (e.g., glass ionomer or composite) to mask staining, or limiting SDF to teeth that are already non-restorable.
- Parental “staining fatigue”—unlike the previously described “SDF fatigue” (Section 6.1), some caregivers accept initial staining but later request removal or replacement, even if the lesion remains arrested. This should be anticipated during follow-up planning.

Current guidelines (ADA, AAPD) acknowledge staining as a limitation but do not provide detailed protocols for aesthetic triage [2,3,4,5,6]. The preservation-to-precision framework addresses this gap by including “acceptable psychosocial impact” as a core child-centered endpoint, which for many families includes aesthetic acceptability.

## 6. Ethical and Systems Dimensions of Interim Stabilization

### 6.1. The Ethical Boundary: Bridge vs. Destination

Interim stabilization is ethically defensible when used as a time-limited strategy with defined goals (e.g., arresting lesions, reducing near-term risk, enabling acclimatization, securing referral) and when there is a credible plan to achieve durable health outcomes. Treatment becomes ethically precarious when it effectively shifts the child into a prolonged ‘managed disease’ state, especially if symptoms persist, progression continues, or follow-up is not feasible.

A rights-informed lens, drawn from the UN Convention on the Rights of the Child (CRC), can complement traditional clinical ethics by emphasizing the child’s best interests and the right to the highest attainable standard of health [18]. Although the United States has not ratified the CRC, its principles offer a structured way to assess whether the care pathway prioritizes the child’s well-being or normalizes prolonged exposure to disease because of system constraints.

**Assessing psychosocial impact in young children (ages 3–4 years):** Direct self-reports are unreliable. Clinicians should use validated parent-proxy instruments, including the Early Childhood Oral Health Impact Scale (ECOHIS) [19] and the Pediatric Quality of Life Inventory (PedsQL) Oral Health Scale [20]. These tools assess domains such as child pain, irritability, sleep disruption, eating difficulties, and parental distress. A practical clinical approach is to ask two standardized questions at each recall: (1) “Has your child complained of tooth pain or avoided any foods in the past month?” and (2) “Has your child’s sleep been disturbed by dental problems?” Positive responses should prompt a re-evaluation of the interim plan.

**Parental “SDF fatigue” and complacency:** A specific ethical challenge arises when a child remains asymptomatic after SDF application. Caregivers may perceive the problem as resolved and lose motivation to pursue definitive care, turning a planned bridge into an indefinite default. To mitigate this, consent discussions should explicitly state that the absence of symptoms does not equal restored function or prevent long-term breakdown. Written care agreements that specify a maximum duration for interim status (e.g., “This plan is for 6 months only”) and include automatic recall reminders can reduce complacency.

**“Bridge ethics” requires:**

1. **Transparent communication and consent:** Caregivers should understand what SDF is intended to support (lesion arrest) and what it does not guarantee (durable function, aesthetics, or tooth survival without further care).
2. **Defined monitoring and exit criteria:** Plans should specify recall timing and escalation triggers (pain, infection, failure to arrest, functional breakdown) [7].
3. **Proportionality to tooth prognosis and service time:** For teeth expected to function for several years, clinicians should assess whether interim stabilization can realistically preserve function and well-being.
4. **Attention to justice and equity:** Interim stabilization can either reduce inequities (by preventing deterioration when access is delayed) or worsen them (if children with fewer resources are disproportionately left on “bridges” that never lead to definitive care).

### 6.2. Access Constraints in High-Income Settings

In systems where definitive restorative care (including treatment under procedural sedation or GA) is delayed, interim stabilization can shift from a deliberate bridge to an unplanned default. The AAPD has documented the denial of operating room (OR) access for pediatric dental treatment as a growing U.S. problem marked by long waiting times and the deferral of medically necessary care [21,22].

**International comparisons:** In England, NHS data indicate that pediatric dental GA wait times exceed 52 weeks in some regions, suggesting that repeated SDF applications serve as a substitute for care [23]. In Australia, average waiting times for public dental care for children with SECC are 12–18 months, and SDF is often used for prolonged interim management [24]. These examples illustrate that the problem is not unique to North America; it reflects broader systemic challenges in high-income countries.

When definitive pathways are constrained, clinicians may appropriately use SDF to arrest lesions and reduce near-term risk. However, access barriers can create a subtle but consequential shift from “interim stabilization while arranging definitive care” to “interim stabilization because definitive care is not realistically available.” This shift can normalize prolonged exposure to disease as routine rather than as a contingency response.

A preservation-to-precision approach requires that access realities be made explicit in planning, documentation, and caregiver communication. This includes stating whether SDF is being used because it is proportionate to disease stage or because definitive care is delayed, and defining monitoring intervals and exit criteria rather than allowing open-ended temporization.

## 7. A Preservation-to-Precision Pathway for the 3- to 4-Year-Old with SECC

**Figure 1:**
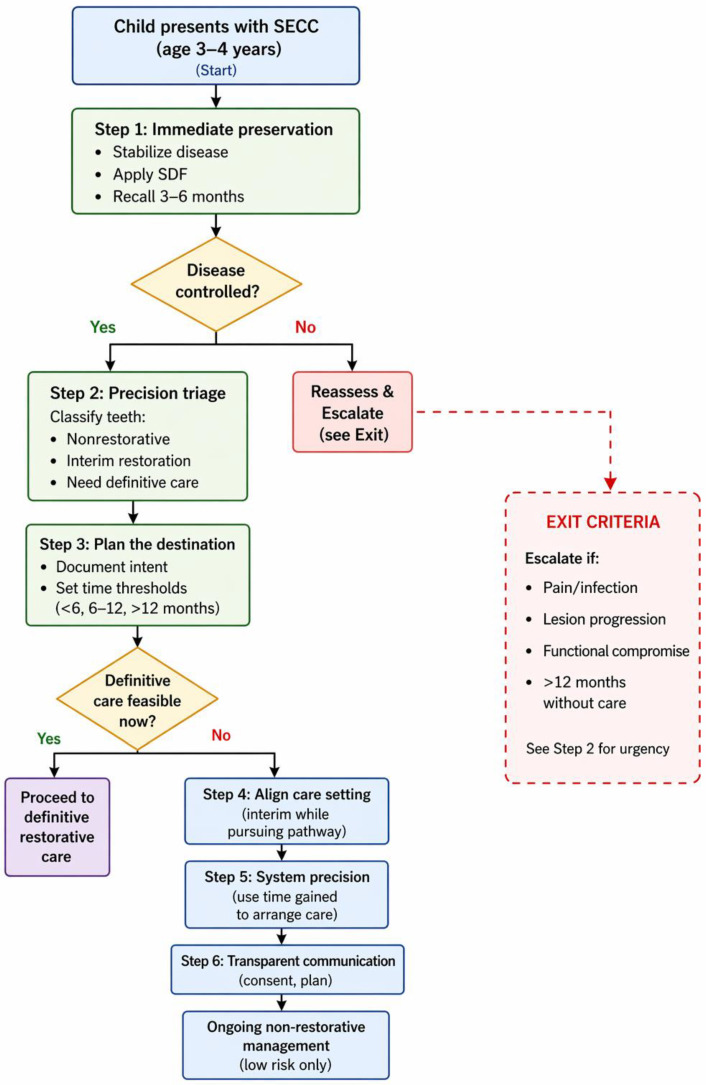
**Preservation-to-Precision pathway flowchart for a 3- to 4-year-old patient with severe early childhood caries (SECC).** The flowchart outlines sequential decision points from initial presentation through interim stabilization and triage to definitive restorative care or ongoing nonrestorative management. The “Exit Criteria” box is explicitly linked to Step 2 (Precision Triage) and includes time thresholds (6 and 12 months) and clinical triggers (pain, infection, functional failure).

The following pathway integrates evidence-based recommendations, staged disease logic, and real-world insights into a structured clinical approach [7]. A visual summary is shown in Figure 1.

**Step 1. Immediate preservation: Stabilize the disease and reduce suffering.**

Define stabilization using child-centered criteria: pain status, signs of infection, functional impact (sleep and eating), and caregiver capacity. Use nonrestorative therapies (including SDF) to arrest lesions within an explicit plan specifying targeted lesions, behavioral guidance, preventive regimens, and reassessment intervals [2,3,4,5,6].

**Step 2. Precision triage: Do not treat SECC as a single entity.**

Classify teeth into categories such as:

- **Appropriate for ongoing nonrestorative management**: Arrestable lesions with realistic follow-up [2].
- **Appropriate for minimally invasive interim restorations**: Structural loss compromises function, but immediate definitive treatment is not feasible [12].
- **Need for timely definitive restorative care**: Teeth with long expected service times, functional demands, or risk profiles where durable restoration is more likely to provide sustained freedom from pain/infection and reliable function [7,12].

Real-world data from Whitlatch et al. [13] underscored the importance of this triage approach: children with severe disease (classes III, IV, V) had escalation rates of 42–50%, whereas those with limited disease (class I) had only a 10% escalation rate.

**Step 3. Plan the destination: Make the bridge explicit.**

When SDF (or any interim strategy) is used, document the following:

- Stated intent (e.g., “interim stabilization while arranging definitive care”);
- Monitoring interval (time-bound follow-up);
- Explicit exit criteria (pain, infection, failure to arrest, functional compromise, caregiver preference, or time thresholds).

**Time-threshold guidance:** Based on available real-world data [13,15,16], we propose the following:

- **Less than 6 months** of planned interim stabilization: generally consistent with “buying time” for access navigation.
- **Between 6–12 months:** requires documented justification and active pursuit of definitive care.
- **Over 12 months without definitive care scheduled: constitutes “selling time”**—prolonged temporization has become de facto treatment. Whitlatch et al. [13] reported that the median time to escalation was 547 days (~18 months) and the median total care duration was 637 days, indicating that many children remain in interim status for more than a year before needing higher-intensity care. This window must be actively managed with exit criteria, not by passive waiting.

**Step 4. Align the care setting with humane, achievable treatment.**

For children for whom chairside definitive care is not feasible, interim stabilization should be used to reduce near-term deterioration while pursuing definitive pathways—not as a substitute for them [16].

**Step 5. System precision: Use “time gained” to move forward.**

When SDF delays sedation/GA by weeks to months, that interval is beneficial only if it is used to advance toward definitive care and to maintain child-centered endpoints during the wait [16]. Access constraints must be made explicit in planning and communication [21,22].

**Step 6. Communicate transparently: Align consent with evidence.**

Caregivers should receive clear explanations of the intended goals of interim stabilization (lesion arrest), what it does not guarantee (durable function/aesthetics/tooth survival without further care), and the follow-up and escalation plan. The Whitlatch et al. [13] experience shows that even with an optimized SOP, many families will need to return for escalation; this should be discussed first.

## 8. What We Should Stop Claiming—And What We Should Start Measuring

### 8.1. Claims to Avoid or Qualify

- Stop treating SDF as a universal long-term protocol for SECC. The guidelines support lesion arrest, not blanket long-term substitution [2,3,4,5,6]. The Whitlatch et al. [13] data show that even in a best-case private practice, 35% of children escalate within ~2 years.
- Stop implying that “buying time” equals “securing outcomes to exfoliation.” Real-world data show substantial subsequent treatment within 2 years for many SDF-treated teeth [13,15].
- Stop presenting pathway changes as evidence of clinical endpoints. Observational findings on utilization patterns do not directly prove reduced pain, fewer infections, or improved quality of life [13,14,15,16,17].
- Stop conflating “minimal invasiveness” with “minimal obligation.” A minimally invasive intervention can be appropriate yet ethically inadequate if it serves as an open-ended substitute for timely definitive care when indicated.

### 8.2. Claims Supported by Evidence

- SDF and other nonrestorative therapies are evidence-based for arresting lesions in primary teeth when used within an ongoing caries management plan [2,3,4,5,6,7,8,9,10,11].
- Conservative restorative approaches are also available for cavitated lesions when durability is required to meet health endpoints [12].
- In real-world settings, SDF often alters care pathways, may delay sedation/GA by weeks to months, and frequently precedes subsequent treatment within two years for a substantial proportion of treated teeth [13,14,15,16,17].
- Severe caries subtypes (multisurface, canine-involving) have escalation rates of 42–50%, justifying precision triage [13].

### 8.3. Research Priorities

- **Tooth-level outcomes in high-burden SECC cohorts:** Survival to exfoliation by tooth type and baseline lesion severity [17].
- **Patient-centered outcomes:** Prospective measures of pain episodes, infection events, sleep disruption, and oral health-related quality of life [1,19,20].
- **Comparative effectiveness of staged pathways:** Interim stabilization plus timely definitive restoration versus prolonged temporization [7,12].
- **Equity outcomes:** The proportion of patients who receive timely definitive care versus those who remain on extended interim management.
- **System outcomes:** Wait times to definitive care, appointment completion rates, sedation/GA capacity, and cost trade-offs [21,22].

## 9. Conclusions

The appropriate role of 38% silver diamine fluoride in severe early childhood caries is best understood as evidence-supported lesion arrest and interim stabilization within an explicit care pathway, rather than as a universal long-term substitute for definitive restorative care. Professional guidance supports SDF for arresting cavitated lesions in primary teeth within an ongoing caries management plan [2,3,4,5,6]. Real-world data from multiple studies—including a large recent private-practice implementation [13]—indicate that many SDF-treated teeth receive additional treatment within about two years, and that any delay in sedation/general anesthesia is typically measured in weeks to months [16]. Furthermore, Whitlatch et al. [13] demonstrated that even under optimized conditions with a standardized disease management protocol, 35% of children with caries escalate to higher-intensity treatment, with severe subtypes showing escalation rates of 42–50% and a median time to escalation of 547 days.

The preservation-to-precision framework offers a practical, ethically grounded approach that prioritizes child-centered outcomes through tooth- and child-specific planning; a realistic appraisal of follow-up feasibility; and predefined exit criteria for escalation when durability, function, or symptom control cannot be reliably maintained. In high-income settings, the value of “buying time” depends on whether systems use it to move children toward timely, definitive care rather than normalizing prolonged temporization as routine practice. Future work should prioritize patient-centered outcomes, equity analyses, and pathway studies that evaluate staged approaches under real-world access constraints.

## Figures and Tables

**Table 1:** Summary of selected real-world studies on SDF in pediatric populations.

Author, Year	Study Design	Follow-Up Duration	Success Criteria Used	Key Finding (SDF Group)	Implication for “Bridge vs. Default”
Davis et al., 2020 [14]	Retrospective matched cohort	1 year	Subsequent treatment events, GA use, and expenditures	SDF group: more visits, fewer restorations, lower expenditures, more frequent GA use over 1 year	SDF alters utilization patterns; may reflect case selection rather than GA prevention
Schlotz et al., 2024 [15]	Retrospective claims analysis	≥24 months	Need for additional treatment (restoration, extraction, repeat SDF)	40% of SDF-treated primary teeth received additional treatment; odds decreased with age	SDF often marks a stage of care, not an endpoint; durable plans should anticipate subsequent intervention
Meyer et al., 2023 [16]	Retrospective cohort	Until sedation/GA claim (median 63–91 days)	Time to first sedation/GA	SDF associated with delay to sedation/GA of 63–91 days depending on provider concordance	SDF may extend interval before sedation/GA; does not prevent sedation/GA or guarantee symptom-free waiting
Raskin et al., 2021 [17]	Naturalistic survival analysis	Up to 24 months	Tooth survival (no follow-on procedure)	Overall survival 76%; varied by tooth type and age	SDF can perform well, but outcomes are heterogeneous; survival endpoints do not capture pain, infection, or quality of life
Whitlatch et al., [13]	Retrospective descriptive cohort	Up to ~7 years (median 1.75 years)	Escalation to higher-intensity treatment (restoration, extraction)	35% of children with caries escalated; median time to escalation 547 days; severe subtypes (Class III, V) had 42–50% escalation	Even with an optimized disease-management SOP, escalation is common within ~1.5–2 years, supporting the need for planned exit criteria rather than open-ended temporization

## Data Availability

No new data were created or analyzed in this study.
